# On the Perils of Universal and Product-Led Thinking: Comment on "How Neoliberalism Is Shaping the Supply of Unhealthy Commodities and What This Means for NCD Prevention"

**DOI:** 10.15171/ijhpm.2019.109

**Published:** 2019-11-16

**Authors:** Clare Herrick

**Affiliations:** Department of Geography, King’s College London, London, UK.

**Keywords:** Neoliberalism, Policy, Noncommunicable Disease, Risk

## Abstract

Lencucha and Thow’s paper offers an important addition and corrective to the burgeoning body of work in public health on the ‘commercial determinants of health’ in the context of non-communicable diseases (NCDs). Rather than tracing the origins of incoherence across policy sectors to the nefarious actions of industry, they argue that we need to be better attuned to the neoliberal ideologies that underpin these policies. In this commentary I explore two aspects of their argument that I find to be problematic: First, the suggestion that neoliberalism itself has some kind of deterministic or explanatory capacity across vastly different social, spatial, economic and political contexts. Second, I explore their concept of ‘product-based NCD risk,’ a perspective that disembodies and detaches risk from the social and structural conditions of their making.

Lencucha and Thow’s paper has a laudable goal: to explore and explain policy (in)coherence – within and between the health and economic sectors – pertaining to what they term ‘product-based non-communicable disease (NCD) risk.’^1^ In the authors’ commentary, such ‘policy incoherence’ refers to the decoupling of economic policy from social and health policy. As they then argue, where the means and ends of health and economic policies deviate, this has often been explained by the ‘nefarious strategies used by industry to promote unhealthy products and prevent their regulation.’^1^ Recently, such research and writing has been undertaken under the frame of the ‘commercial determinants of health.’^2,3^ While there is great truth in the belief that the strategies of big business can be immoral and detrimental to human health, Lencucha and Thow suggest that there is more underpinning the story than the corporate conspiracies that often accompany public health analyses of the commercial determinants of (poor) health.

Their paper argues that ‘the friction that inhibits healthy product policy regimes is the persistence of the neoliberal paradigm in shaping the relationship between government, market and society.’^1^ They further contend that we need to embed ‘products in this broader policy context’^1^ in order to better understand the paradigms that underpin institutional landscapes. At this paper’s core, therefore, is the assertion that in order to understand policy incoherence, we must first appreciate and interrogate the tight grip of the neoliberal policy paradigm across the world and how this, in turn, shapes divergent expectations of the relationship between the state, markets and consumer-citizens. This paradigm, they argue, is a barrier to health and economic policy coherence given that the aspirations of neoliberalism – a free market untethered from state intervention, less Government but more governance, the value of privatisation and a belief in the sanctity of individual autonomy (rather than social responsibility) – are almost always incompatible with the kinds of social and health policies that are needed to ensure universal well-being.

These are undeniably important interventions for those in public health who have become increasingly attuned to seeking out the nefarious rather than questioning the prevailing ideology or ideologies that might undergird and justify this.^4,5^ Stepping back from the front line of these vested interests is also essential in order to start to carve out more nuanced and qualitative engagements with the role that industries play in shaping our health outcomes and how governments and their policy choices might enable or constrain these.^6,7^ However, as Bell and Green’s^8^ recent editorial in *Critical Public Health* should remind us, public health’s often-reductionist tendency to evoke deterministic narratives of neoliberalism as a catch-all explanation for everything that is wrong with the private or public sectors’ approach to health often lacks the spatial and social nuance, as well as qualitative depth, that many social scientists have argued for. This marks the first of my two avenues of critique of this paper.

Lencucha and Thow’s assertion that, first, neoliberalism has become ‘the dominant paradigm’^1^ and that second, this ‘has conditioned the policy environment in a way that promotes the supply of unhealthy commodities’^1^ is to fall into the trap identified by Ward and England^9^ of casting neoliberalism as an ideological hegemon. Here I argue that, in keeping with the arguments made by Brenner and Theodore,^10^ neoliberal projects are not only multiple, they are also embedded within particular geographic, social and economic contexts which, in turn, condition the particularities of their emergence and deployment. To state the obvious, while both France and the United States are neoliberal *economies,* the social contract between citizens and government is vastly different in each. As such, they do not share a universal conception or ideology of the relationship between the state, market and citizens. This is especially the case in the domain of health. And, while Lencucha and Thow clearly accept that the neoliberal paradigm is not uniformly applied, they do insist that, as a ‘dominant’ political, social and economic ideology, its uptake holds huge explanatory weight. As they further suggest, if this is acknowledged and understood by health advocates, then they can be ‘more sympathetic to policy-makers’^1^ across the sectors that are often so heavily critiqued within the commercial determinants of health mindset.

My concern is that such reductionism is largely unrepresentative of the vastly different ways in which health plays into and is an outcome of the market-state-citizen nexus across the world. Tracing policy incoherence to neoliberal ideology also lends credence to the belief that there can be universal ‘best-buys’ or policy-solutions to solve the world’s NCD woes.^11^ Yet, there is no one neoliberal paradigm, no one set of neoliberal policies and certainly no unifying experience of living in a neoliberal society. The suggestion that the dynamic intricacies of ‘policy incoherence’ are a ‘logical extension to’^1^ the tentacles of neoliberal ideology supports the assertion that ‘evidence-based’ best buys can be unproblematically transferred across the world. Yet, as Brenner and Theodore make clear, ‘the global imposition of neoliberalism has been highly uneven, both socially and geographically and its institutional forms and sociopolitical consequences have varied significantly across spatial scales.’^10^ Rather than seeking the out the explanatory power of a ‘dominant ideology,’ greater attention is needed to the social, economic and political contexts that help explain why the application of best buys can often have unintended consequences.

To talk or write of neoliberalism as if it was a coherent and universally-agreed ideology with a clearly demarcated channel to policy outcomes is, I suggest, to thoroughly miss its ‘polycentric and multiscalar character.’^10^ It is important to remember that citizens (whose voices are often neglected by Luncucha and Thow) can also have hugely divergent expectations of individual liberty and freedom to consume ‘unhealthy’ products within neoliberal economies. Reducing policy incoherence to a singular neoliberal paradigm offers partial insight, but my fear is that it also reinforces a public health tendency to talk about the world in vastly generalised terms. For example, the example used in the paper that rolls together alcohol policy across the vastly different countries of Lesotho, Uganda, Malawi, and Botswana^12^ misses an important counter-factual – that Botswana’s 70% alcohol levy and tight regulation of traditional beer depots was decidedly *anti-neoliberal* in the state’s regulatory approach, much to the dismay of liquor producers and retailers.^13^ This example is but one, but serves to problematise any assertion that there can ever be a ‘logical extension of the neoliberal paradigm.’^1^ Instead, we see only the ‘bricolage’ of policy experiments and failures that may be underpinned to a greater or lesser extent by a series of (often inconsistent and illogical) views of how the state, market and society should interrelate.^14^

My second line of critique follows Lencucha and Thow’s fascinating use of the term ‘product-based NCD risk.’ It is clear that many products are bad for our health – alcohol, ultra-processed foods, too much salt, tobacco – yet, there is something about appending risk to the product alone that, as a social scientist, I find curious. Conjoining the terms ‘product-based NCD risk’ suggests that products alone are a risk factor for NCDs or that the aetiology of NCDs can be traced to products. Yet, products are purchased and consumed in contexts and environmental settings that far outstrip the capacity of the product alone to be harmful. For example, while alcohol consumption in the United Kingdom may be highest amongst professional classes, alcohol-attributable harms are highest amongst those of lower socioeconomic status: the ‘alcohol harm paradox.’^15^ The authors do also consider ‘the management of product environments,’^1^ but this term itself is equally restrictive and reductive. Indeed, just as neoliberalism is produced in and through certain socio-spatial contexts, the risk of developing a chronic disease cannot be reduced to a set of (harmful) products and ‘product environments.’ This is especially so as the term ‘product environment’ reduces the nature of the ‘environment’ to product formulation, marketing strategies and corporate efforts to evade regulation rather than the broad socio-ecological contexts more often associated with the term. To focus attention on the ‘product environment’ is thus, ironically, to miss the actual environment in all its holistic complexity.

The perils of ascribing risk to individual or group lifestyles in terms of associated stigma or discrimination are well-documented across infectious diseases^16^ and NCDs.^17^ Yet, theorising risk in terms of products – as often the case within the expanding research domain of the corporate determinants of health – also comes with its own moral perils. While the frame ably draws attention to the nefarious actions of industry, it can also have a flipside. For example, the demonisation of certain products within public health discourse and policy (ie, high-strength cider within the alcohol minimum pricing debate in the United Kingdom) inevitably stigmatises those who choose to consume it and can legitimise a silence on the broader drivers of these practices by prioritising the elimination of demand for the product. Attention to the wrongdoings of industry, the policy paradigms that may support these and their health consequences is essential. However, this should not be at the expense of drawing attention to the broader structural and social conditions that shape the contexts within which buying (or not buying) ‘unhealthy commodities’^18^ takes place. Doing so helps illuminate why ‘product-based NCD risk’ is never universally borne and why critical attention to environments other than those circumscribed by products alone needs to be far more wide-ranging.

The conversations about health risk, systems and beliefs that Lencucha and Thow are initiating through their paper are crucial. Similarly, their efforts to draw attention to the underlying systems of thought that guide a consistent and frustrating state of policy incoherence are long overdue within the public health field. That economic and health or social policies are at odds should come as no shock given the sacrifices expected of the social realm in the name of economic growth and productivity. Yet, focussing attention on ‘the product environment’ in relation to NCDs may inadvertently provide even more of a role for industry: it sanctions product reformulation, revised marketing strategies or entirely new ‘better for you’ products. Indeed, exploring neoliberalism through products in the context of health may further disembed and decontextualise the social issues of real importance. With political horizons as short-term as our individual risk-horizons, the ‘crisis’ of NCDs will only continue to grow. There are many things to blame for this and neoliberal ideology is but one. Within public health, it is important to avoid the temptation to suggest that it is *the* one.^8^

## Ethical issues

Not applicable.

## Competing interests

Author declares that she has no competing interests.

## Author’s contribution

CH is the single author of the paper.

## References

[1] Lencucha R, Thow AM (2019). How neoliberalism is shaping the supply of unhealthy commodities and what this means for NCD prevention. Int J Health Policy Manag.

[2] McKee M, Stuckler D (2018). Revisiting the corporate and commercial determinants of health. Am J Public Health.

[3] Kickbusch I, Allen L, Franz C (2016). The commercial determinants of health. Lancet Glob Health.

[4] Moodie R, Stuckler D, Monteiro C (2013). Profits and pandemics: prevention of harmful effects of tobacco, alcohol, and ultra-processed food and drink industries. Lancet.

[5] Reubi D (2016). Of neoliberalism and global health: human capital, market failure and sin/social taxes. Crit Public Health.

[6] Herrick C (2016). Alcohol, ideological schisms and a science of corporate behaviours on health. Crit Public Health.

[7] Green J (2019). Time to interrogate corporate interests in public health?. Crit Public Health.

[8] Bell K, Green J (2016). On the perils of invoking neoliberalism in public health critique. Crit Public Health.

[9] Ward K, England K. Introduction: Reading Neoliberalization. In: Neoliberalization: States, Networks, Peoples. Wiley; 2007. p. 1-22. 10.1002/9780470712801.ch1

[10] Brenner N, Theodore N (2002). Cities and the geographies of “actually existing neoliberalism. ” Antipode.

[11] World Health Organisation (WHO). From burden to “best buys”: reducing the economic impact of NCDs in low- and middle-income countries. Geneva: WHO; 2011.

[12] Bakke Ø, Endal D (2010). Vested interests in addiction research and policy alcohol policies out of context: drinks industry supplanting government role in alcohol policies in sub-Saharan Africa. Addiction.

[13] Herrick C (2016). Global health, geographical contingency, and contingent geographies. Ann Am Assoc Geogr.

[14] Cleaver F. Development Through Bricolage: Rethinking Institutions for Natural Resource Management. London: Routledge; 2017.

[15] Katikireddi SV, Whitley E, Lewsey J, Gray L, Leyland AH (2017). Socioeconomic status as an effect modifier of alcohol consumption and harm: analysis of linked cohort data. Lancet Public Health.

[16] Farmer P. Infections and Inequalities: The Modern Plagues. Berkeley: University of California Press; 1999.

[17] Seeberg J, Meinert L (2015). Can epidemics be noncommunicable? reflections on the spread of ‘noncommunicable’ diseases. Med Anthropol Theory.

[18] Stuckler D, McKee M, Ebrahim S, Basu S (2012). Manufacturing epidemics: the role of global producers in increased consumption of unhealthy commodities including processed foods, alcohol, and tobacco. PLoS Med.

